# Hepatic transcriptome analysis of inter-family variability in flesh n-3 long-chain polyunsaturated fatty acid content in Atlantic salmon

**DOI:** 10.1186/1471-2164-13-410

**Published:** 2012-08-20

**Authors:** Sofia Morais, John B Taggart, Derrick R Guy, J Gordon Bell, Douglas R Tocher

**Affiliations:** 1 Institute of Aquaculture, University of Stirling, Stirling, FK9 4LA, Scotland, UK; 2 Landcatch Natural Selection Ltd, Cooperage Way, Alloa, FK10 3LP, Scotland, UK

## Abstract

**Background:**

Genetic selection of Atlantic salmon families better adapted to alternative feed formulations containing high levels of vegetable ingredients has been suggested to ensure sustainable growth of aquaculture. The present study aimed to identify molecular pathways that could underlie phenotypic differences in flesh n-3 long-chain polyunsaturated fatty acid (LC-PUFA) levels when fish are fed vegetable oil diets. Liver transcriptome was analyzed and compared in four families presenting higher or lower n-3 LC-PUFA contents at two contrasting flesh total lipid levels.

**Results:**

The main effect of n-3 LC-PUFA contents was in the expression of immune response genes (38% of all significantly affected genes), broadly implicated in the modulation of inflammatory processes and innate immune response. Although genetic evaluations of traits used in the breeding program revealed that the chosen families were not balanced for viral disease resistance, this did not fully explain the preponderance of immune response genes in the transcriptomic analysis. Employing stringent statistical analysis no lipid metabolism genes were detected as being significantly altered in liver when comparing families with high and low n-3 LC-PUFA flesh contents. However, relaxing the statistical analysis enabled identification of potentially relevant effects, further studied by RT-qPCR, in cholesterol biosynthesis, lipoprotein metabolism and lipid transport, as well as eicosanoid metabolism particularly affecting the lipoxygenase pathway. Total lipid level in flesh also showed an important effect on immune response and 8% of significantly affected genes related to lipid metabolism, including a fatty acyl elongase (*elovl2*), an acyl carrier protein and stearoyl-CoA desaturase.

**Conclusions:**

Inter-family differences in n-3 LC-PUFA content could not be related to effects on lipid metabolism, including transcriptional modulation of the LC-PUFA biosynthesis pathway. An association was found between flesh adiposity and n-3 LC-PUFA in regulation of cholesterol biosynthesis, which was most likely explained by variation in tissue n-3 LC-PUFA levels regulating transcription of cholesterol metabolism genes through *srebp2*. A preponderance of immune response genes significantly affected by n-3 LC-PUFA contents could be potentially associated with disease resistance, possibly involving anti-inflammatory actions of tissue n-3 LC-PUFA through eicosanoid metabolism. This association may have been fortuitous, but it is important to clarify if this trait is included in future salmon breeding programmes.

## Background

Aquaculture is the fastest growing animal production activity worldwide, supplying an increasing proportion of fish for human consumption, estimated at around 50% of total supply in 2008 [1]. However, the growth of marine aquaculture is threatened by its excessive reliance on fishmeal (FM) and fish oil (FO) from wild stocks for the production of fish feeds, which is also an ecologically unsound practice. Almost 89% of the total global production of FO is currently used by aquaculture [2] and the future of this activity strongly depends on the reduction of dependency on FM and FO and its replacement with alternative ingredients, such as vegetable oils (VO) and plant meals, while maintaining fish welfare and health benefits for the human consumer. Fish are highly nutritious components of the human diet and the main source of essential n-3 long-chain polyunsaturated fatty acids (LC-PUFA). The beneficial effects of fatty acids, such as eicosapentaenoic acid (EPA) and docosahexaenoic acid (DHA), are numerous and important, including protection against a range of cardiovascular and inflammatory diseases, as well as neurological disorders [3]. Atlantic salmon (*Salmo salar*) can grow well on diets where FO has been completely replaced by VO but this results in lower levels of n-3 LC-PUFA in their flesh, compromising their nutritional value and health-promoting effects to the human consumer [4].

The use of selective breeding programs to enhance traits of commercial importance is becoming increasingly common in aquaculture [5]. It has been suggested that combining genetic selection for fish that are more efficient in retaining and/or biosynthesising n-3 LC-PUFA with changes in commercial diet formulations (i.e., higher levels of inclusion of VO) might be a viable strategy to meet growing worldwide demands for aquaculture products, without loss of nutritional value. Previous studies have shown wide individual variability in the capacity of Atlantic salmon to retain or synthesize n-3 LC-PUFA when fed VO diets [6]. Following this, Leaver et al. [7] demonstrated that deposition and/or retention in flesh of dietary n-3 LC-PUFA, EPA and DHA, is a highly heritable trait (h^2^ = 0.77) in salmon. These results have prompted further interest in large-scale in-depth studies exploring genotype × nutrient interactions in salmon, analysing whether the genetic background of the fish could affect the physiological response to complete dietary replacement of FO by VO [8,9]. In the present study we investigated this further by analyzing the transcriptome from liver, the primary site of synthesis and export of lipids to extra-hepatic tissues including flesh, from four Atlantic salmon families phenotyped for different levels of flesh n-3 LC-PUFA content in response to a VO diet. The objective was to identify gene pathways and molecular mechanisms that might underlie differences in flesh n-3 LC-PUFA contents when salmon families were fed the same low LC-PUFA diet. Furthermore, because n-3 LC-PUFA level is a component of, and associated with total lipid content in a tissue, a factorial design was chosen in which families containing higher and lower proportions of flesh n-3 LC-PUFA were compared at similar flesh total lipid contents.

## Results

### Family lipid contrasts

Lipid analysis of fifty Atlantic salmon families showed flesh lipid levels ranging from 2.3 to 5.7% of wet weight, with relative and absolute n-3 LC-PUFA contents varying from 71 to 136 (μg/mg lipid) and 314 to 554 (mg/100 g flesh), respectively. As expected, high correlations between lipid level and n-3 LC-PUFA content were observed (r = −0.65 or 0.70 for relative and absolute contents, respectively), indicating that only families with near identical lipid levels should be compared to avoid confounding effects associated with the lipid level factor (additional file 1). Using these results, four families were identified; two with high (H) levels of lipid (5 g/100 g flesh), and two with low (L) levels of lipid (3.5 g/100 g flesh) and, within each level of total lipid, the two families had significantly contrasting relative n-3 LC-PUFA contents (similarly termed H and L). Therefore, the four families constituted a 2 x 2 factorial design, labelling each family by the total lipid/n-3 LC-PUFA contrasts as LL, LH, HL and HH, respectively (Table 1), which allowed comparisons of n-3 LC-PUFA contents at a constant lipid level and, similarly, comparisons of total lipid at constant n-3 LC-PUFA levels.

**Table 1:** Lipid phenotypes of families chosen for molecular analysis

**Family**	**Total Lipid (g/100 g flesh)**	**n-3 LC-PUFA**		**ARA**	
		**Relative (μg/mg lipid)**	**Absolute (mg/100 g flesh)**	**Relative (μg/mg lipid)**	**Absolute (mg/100 g flesh)**
LL	3.5 ± 0.4	105.1 ± 3.8 *	363.0 ± 30.3	3.45 ± 0.13 *	11.93 ± 1.00
LH	3.5 ± 0.7	133.8 ± 4.8 *	468.0 ± 92.9	4.25 ± 0.06 *	14.87 ± 2.94
HL	5.1 ± 0.8	83.7 ± 14.0 *	426.9 ± 103.2	2.70 ± 0.53	13.81 ± 3.80
HH	5.0 ± 0.7	112.0 ± 7.9 *	554.3 ± 50.7	3.67 ± 0.63	18.04 ± 2.09

Indicated are levels of total lipid (g/100 g flesh, wet weight), and relative and absolute contents of total n-3 LC-PUFA and of the n-6 LC-PUFA arachidonic acid (ARA) in the flesh (n = 3 pools) of the 4 Atlantic salmon families used in the transcriptomic analysis. Asterisks signify significant differences between the two families with the same total lipid content.

### Microarray analysis

A two-way ANOVA analysis employing the Benjamini-Hochberg multiple testing correction (at a significance level of 0.05 and fold change cut-off of 1.2) was performed to assess significant effects of the factors 'n-3 LC-PUFA' and 'total lipid', which returned lists with 43, 109 and 66 entities for each factor and their interaction, respectively. These significant lists were then analyzed in detail and genes were categorized according to their biological function, in some cases inferred from mammalian homolog genes (Tables 2 and 3). Because the focus of this work was to identify genes that are specifically affected by the trait n-3 LC-PUFA content without the interference of total lipid level, the interaction between the two factors is not presented. Distribution of genes by categories of biological function (excluding 12-18% non-annotated probes, those representing the same gene or with a miscellaneous function) revealed that there was a preponderance of immune response genes significantly affected by both factors: 38% by 'n-3 LC-PUFA' and 29% by 'total lipid'. Gene Ontology (GO) enrichment analysis, which enables the identification of GO terms significantly enriched in the input entity list when compared to the whole array dataset, revealed that this is a true over-representation in the list of genes significantly affected by the 'total lipid' factor (Additional file 2). In contrast, genes involved in the broad category of metabolism only corresponded to 21% of genes significantly affected by n-3 LC-PUFA content and 30% by the 'total lipid' factor. Surprisingly, no lipid metabolism genes were significantly altered in liver when comparing families with higher and lower contents of n-3 LC-PUFA in their flesh, while about 8% were significantly affected by flesh lipid level. Within these, noteworthy was the down-regulation of fatty acyl elongase (*elovl2*) and of acyl carrier protein transcripts in salmon having a higher lipid level in their flesh, independent of LC-PUFA content. On the other hand, stearoyl-CoA desaturase was significantly up-regulated in fish with higher lipid levels in their flesh. The interaction between both factors is not presented but it did not substantially affect lipid metabolism genes. Finally, and in general, genes involved in regulation of transcription and signalling were also prevalent, 17% in response to 'n-3 LC-PUFA' and 12-13% to 'total lipid'.

**Table 2:** Liver transcripts differentially expressed when examining the explanatory power of the factor 'n-3 LC-PUFA' content in flesh of four families of Atlantic salmon fed the same low FM/high VO diet

**Probe name**	**Gene**	**High/Low LC-PUFA**		**p-value**
		**L Lipid (LH/LL)**	**H Lipid (HH/HL)**	
***Metabolism (21%)***				
*Energy metabolism (4%)*				
Ssa#S31995754	Cytochrome c oxidase subunit 2	7.1	- 1.0	0.0001
*Protein and amino acid metabolism (13%)*				
Ssa#CB502423	N-acetylated alpha-linked acidic dipeptidase-like 1	3.7	79.8	0.0011
Ssa#STIR03710	Proteasome subunit beta type-9 precursor	- 14.9	- 1.1	0.0026
Ssa#S31993738_S	Ubiquitin-conjugating enzyme E2	- 3.3	- 1.4	0.0188
*Xenobiotic and oxidant metabolism (4%)*				
Ssa#S18892279	Cytochrome P450 1A	1.8	1.4	0.0096
Ssa#STIR00161_2	Cytochrome P450 1A	1.9	1.4	0.0160
Ssa#STIR00161_3	Cytochrome P450 1A	2.5	1.8	0.0213
Con_CANDS_13	Cytochrome P450 1A	2.0	1.4	0.0494
***Translation (8%)***				
Ssa#STIR26031	Mitochondrial 28 S ribosomal protein S34	- 11.1	1.5	0.0017
Ssa#S18867312	Ribonuclease UK114	1.4	1.4	0.0450
***Regulation of transcription (17%)***				
Ssa#S35510106	Zinc finger protein 367	- 1.4	6.5	0.0026
Omy#S18104058	Zinc finger protein 235	4.1	2.3	0.0058
Ssa#TC111702	Reverse transcriptase-like protein	- 1.3	4.6	0.0104
Ssa#TC112002	Retinoid X receptor beta	1.0	- 19.1	0.0134
***Signalling and protein modification (17%)***				
Ssa#STIR15776	Dolichyl-diphosphooligosaccharide-protein glycosyltransferase subunit 4	- 1.7	123.3	0.0000
Ssa#STIR23530	Dolichyl-diphosphooligosaccharide-protein glycosyltransferase subunit 4	- 1.9	115.6	0.0000
Ssa#STIR03642	Dolichyl-diphosphooligosaccharide-protein glycosyltransferase subunit 4	- 1.7	68.1	0.0000
Ssa#STIR01857	Dolichyl-diphosphooligosaccharide-protein glycosyltransferase subunit 4	- 1.5	125.5	0.0001
Ssa#STIR31840	Sphingomyelin phosphodiesterase acid transcript variant 1	2.1	2.3	0.0303
Ssa#STIR07369	RAF1 proto-oncogene serine/threonine-protein kinase	1.8	1.3	0.0343
Ssa#S35552908	Tyrosine 3-monooxygenase/tryptophan 5-monooxygenase activation protein, epsilon polypeptide	- 1.6	- 2.1	0.0455
***Immune response (38%)***				
Ssa#S35536179	similar to novel NACHT domain containing protein	2.4	1.8	0.0026
Ssa#S35516341	Tripartite motif-containing protein 25 (*trim25*)	5.2	6.7	0.0066
Ssa#S30241035	MHC class I	- 1.3	- 26.0	0.0079
Ssa#STIR02298	c-c motif chemokine 13 precursor (*ccl13*)	4.4	3.0	0.0100
Ssa#S35581943	Myelin and lymphocyte protein (*mal*)	- 22.2	- 25.0	0.0100
Ssa#KSS3969	Leukocyte cell-derived chemotaxin 2 precursor (*lect2*)	4.6	7.1	0.0134
Ssa#STIR15577	Tissue factor pathway inhibitor a	22.3	1.7	0.0135
Omy#S15332652	Pentraxin	- 1.1	199.4	0.0172
Ssa#TC70262	Cathepsin K	1.4	2.3	0.0279
Ssa#S35558945	Tripartite motif-containing protein 25 (*trim25*)	4.8	6.5	0.0347
***Miscellaneous/unknown function***				
Ssa#STIR09736	Transmembrane protein 42	2.3	1.9	0.0009
Ssa#S35519407	Tetratricopeptide repeat protein 23	3.7	1.0	0.0011
Ssa#STIR02307	Family with sequence similarity member a (FAM36A)	1.3	3.6	0.0026
Omy#BX309274	X-ray repair complementing defective repair in Chinese hamster cells 3	- 1.0	- 8.0	0.0100
Ssa#STIR21287	Solute carrier family 30 (zinc transporter) member 7	2.1	1.5	0.0100
Ssa#S35521859_S	Family with sequence similarity member a (FAM36A)	- 1.1	4.8	0.0358
Ssa#S18842295	Alveolin	1.7	4.6	0.0422

Results were obtained by two-way ANOVA analysis (p < 0.05; fold change cut-off of 1.2) with Benjamini-Hochberg multiple testing correction. Transcripts (43 probes; 12% unknowns) are arranged by categories of biological function and, within these, by p-value. Indicated are also the probe names and the expression ratio determined separately between families with high vs low n-3 LC-PUFA contents, for each total lipid level. Percentages of distribution of genes within each category do not include non-annotated probes, those representing the same gene or with a miscellaneous function.

**Table 3:** Liver transcripts differentially expressed when examining the explanatory power of the variable 'total lipid' level in flesh of four families of Atlantic salmon fed the same low FM/high VO diet

**Probe name**	**Gene**	**High/Low Lipid**		**p-value**
		**Lo LC-PUFA (HL/LL)**	**Hi LC-PUFA (HH/LH)**	
***Metabolism (30%)***				
*Lipid metabolism (8%)*				
Ssa#STIR02479	Butyrophilin subfamily 2 member A2 precursor	7.76	1.23	0.0001
Ssa#STIR03356	Acyl carrier protein, mitochondrial precursor	- 5.17	- 1.55	0.0004
Ssa#STIR00151_3	Polyunsaturated fatty acid elongase (*elovl2*)	- 1.40	- 1.87	0.0190
Ssa#STIR00151_2	Polyunsaturated fatty acid elongase (*elovl2*)	- 1.28	- 1.97	0.0238
Con_CANDS_03	Polyunsaturated fatty acid elongase (*elovl2*)	- 1.33	- 1.82	0.0310
Ssa#STIR24266	Acyl carrier protein, mitochondrial precursor	- 2.88	- 1.52	0.0312
Ssa#STIR21802	Stearoyl-CoA desaturase	3.53	3.17	0.0324
Ssa#STIR00151_4	Polyunsaturated fatty acid elongase (*elovl2*)	- 1.41	- 1.82	0.0393
Ssa#S35501441_S	Acyl carrier protein, mitochondrial precursor	- 2.65	- 1.44	0.0457
Ssa#KSS4634	Stearoyl-CoA desaturase	3.52	2.82	0.0486
*Energy metabolism and generation of precursor metabolites (11%)*				
Ssa#S31995754	Cytochrome c oxidase subunit 2	6.57	- 1.12	0.0002
Ssa#STIR03496	LYR motif-containing protein 5	1.20	1.48	0.0233
Ssa#STIR03740	6-Phosphogluconate dehydrogenase, decarboxylating	1.60	1.93	0.0238
Ssa#TC106663	Creatine kinase, testis isozyme	1.76	1.46	0.0380
Ssa#STIR19155	NADH dehydrogenase iron-sulfur protein 7	1.21	2.30	0.0409
Ssa#STIR12872	V-type ATPase B subunit	- 1.19	- 1.24	0.0419
*Protein and amino acid metabolism (4%)*				
Ssa#STIR03710	Proteasome subunit beta type-9 precursor	1.94	25.52	0.0001
Ssa#S30294677	Serine protease HTRA1 precursor (Serine protease 11)	3.42	2.44	0.0089
*Carbohydrate metabolism (4%)*				
Ssa#TC106766	Glycogenin	8.64	1.67	0.0093
Ssa#S30290426	Serine dehydratase-like	1.60	1.72	0.0393
*Xenobiotic and oxidant metabolism (4%)*				
Ssa#S35671757	Extracellular superoxide dismutase	2.18	1.09	0.0061
Ssa#STIR25620	Microsomal glutathione S-transferase 1	2.45	2.72	0.0096
Omy#S18159333	Microsomal glutathione S-transferase 1	2.47	3.15	0.0233
***Transport (2%)***				
Ssa#S35599996	ATPase, H + transporting, lysosomal, V1 subunit H	45.10	15.16	0.0000
***Translation (4%)***				
Ssa#STIR26031	Mitochondrial 28 S ribosomal protein S34	1.19	19.22	0.0000
Ssa#S30241612	39 S ribosomal protein L16	1.30	1.51	0.0324
***Regulation of transcription (12%)***				
Ssa#STIR06878	Cytosolic iron-sulfur protein assembly 1	- 1.16	- 1.56	0.0106
Ssa#TC112002	Retinoid X receptor beta	1.20	- 16.00	0.0254
Omy#S15320037	SWI/SNF-related matrix-associated actin-dependent regulator of chromatin a4	- 1.97	- 1.53	0.0324
Ssa#CN181280	alpha thalassaemia mental retardation X-linked protein	- 3.05	- 6.90	0.0419
Ssa#S35697153	YLP motif containing 1	- 1.49	- 1.32	0.0428
Ssa#S35486480	Zinc finger protein 492	- 1.08	- 2.29	0.0452
***Signalling and protein modification (13%)***				
Ssa#STIR15776	Dolichyl-diphosphooligosaccharide-protein glycosyltransferase subunit 4	- 84.27	2.44	0.0000
Ssa#STIR23530	Dolichyl-diphosphooligosaccharide-protein glycosyltransferase subunit 4	- 83.34	2.60	0.0000
Ssa#STIR03642	Dolichyl-diphosphooligosaccharide-protein glycosyltransferase subunit 4	- 49.76	2.34	0.0001
Ssa#STIR01857	Dolichyl-diphosphooligosaccharide-protein glycosyltransferase subunit 4	- 74.19	2.51	0.0011
Ssa#S35516167	14 kDa phosphohistidine phosphatase	1.57	1.34	0.0019
Ssa#STIR11086	Lunatic fringe	- 5.47	- 2.00	0.0154
Ssa#S30263209	HtrA serine peptidase 3	3.71	2.33	0.0154
Ssa#STIR22920	HCLS1-associated protein X-1	1.73	2.33	0.0390
Ssa#S35701148	Phosphatase and actin regulator 3	- 1.52	- 65.53	0.0404
Omy#S15290792	Serine/threonine-protein kinase PLK2	1.70	2.03	0.0468
***Immune response (29%)***				
Ssa#STIR00130_4	Complement factor H precursor (*cfh*)	- 1.43	- 1.67	0.0012
Ssa#S35536179	novel NACHT domain containing protein	- 1.89	- 2.41	0.0013
Ssa#S30241035	MHC class I	- 1.55	- 30.59	0.0024
Ssa#S35516341	Tripartite motif-containing protein 25 (*trim25*)	- 5.64	- 4.34	0.0125
Ssa#S18834140	Complement factor H precursor (*cfh*)	- 1.34	- 1.57	0.0131
Omy#utu04b09	Complement factor H precursor (*cfh*)	- 1.56	- 1.70	0.0134
Ssa#STIR15577	Tissue factor pathway inhibitor a	21.03	1.59	0.0151
Ssa#S35558236	C-type lectin domain family 16, member A	4.59	2.25	0.0190
Ssa#STIR10409	CD83 antigen precursor	1.98	1.68	0.0238
Omy#S15332652	Putative pentraxin	- 1.33	159.84	0.0246
Ssa#S35685271	GTPase IMAP family member 7	−168.44	- 4.09	0.0263
Ssa#S35551959	Duodenase-1	- 2.94	- 2.77	0.0349
Ssa#S35558945	Tripartite motif-containing protein 25 (*trim25*)	- 5.74	- 4.28	0.0373
Ssa#S35685273	Lactose-binding lectin l-2 precursor putative	1.56	4.04	0.0380
Ssa#S31977617	Scavenger receptor cysteine-rich type 1 protein m130	- 1.31	- 2.05	0.0404
Ssa#STIR04893	Lactose-binding lectin l-2 precursor putative	1.57	4.15	0.0419
Ssa#S30264865	Indoleamine-pyrrole 2,3-dioxygenase	2.83	1.06	0.0444
Ssa#S31981622	Granzyme A	- 5.31	- 4.33	0.0444
Ssa#S35685718	CD83 antigen precursor	2.03	1.58	0.0462
Ssa#KSS3969	Leukocyte cell-derived chemotaxin 2 precursor (*lect2*)	3.16	4.86	0.0486
***Structural proteins (10%)***				
Ssa#STIR03004	Troponin I, slow skeletal muscle	- 202.28	- 135.03	0.0000
Ssa#CK891024	Vitelline envelope protein gamma	1.07	- 15.43	0.0002
Ssa#STIR02053	Troponin I, slow skeletal muscle	- 70.21	- 10.07	0.0005
Omy#S34312003	similar to Titin (Connectin)	- 44.29	- 29.86	0.0013
Omy#S15317515	Type I keratin E7	5.08	2.43	0.0167
Ssa#STIR05140	Troponin I, slow skeletal muscle	- 13.16	- 14.20	0.0124
Ssa#STIR08802	Myosin regulatory light chain 2, smooth muscle isoform	1.55	1.34	0.0324
***Miscellaneous/unknown function***				
Ssa#S35519407	Tetratricopeptide repeat protein 23	3.64	1.02	0.0012
Ssa#S35584894	NCK-associated protein 1-like	- 1.82	- 1.70	0.0019
Ssa#S35521859_S	FAM36A	1.11	5.75	0.0047
Ssa#STIR02307	FAM36A	1.16	3.31	0.0058
Ssa#TC87798	Envelope polyprotein	- 1.83	- 1.03	0.0233
Ssa#STIR20319	TPA-induced transmembrane protein	- 1.09	1.75	0.0233
Ssa#TC110493_S	Beta-3-galactosyltransferase	6.80	1.80	0.0233
Ssa#S30270166	Transmembrane protein 37	2.13	1.90	0.0254
Ssa#STIR08658	Tetraspanin-3 putative	- 3.20	- 1.47	0.0312
Ssa#S30293470	Deoxyribonuclease gamma precursor	2.19	2.85	0.0366
Ssa#S35667723	Dynein, cytoplasmic 1, light intermediate chain 2	- 1.90	- 1.48	0.0366
Ssa#CX354464	Retinol dehydrogenase 12	2.37	2.35	0.0373
Ssa#S35582016	Type I iodothyronine deiodinase	1.51	1.74	0.0380
Ssa#S35515630	C7orf57	1.32	1.13	0.0380
Ssa#STIR15617	FAM36A	1.02	4.60	0.0403
Ssa#STIR31448	osteopontin-like	- 4.84	- 1.58	0.0419
Ssa#STIR26263	Anterior gradient protein 2 homolog precursor	3.72	1.68	0.0419
Ssa#TC65497_S	Adipocyte plasma membrane-associated protein	2.50	1.64	0.0419
Ssa#TC93681	Ring finger protein 44	- 1.23	- 1.17	0.0455
Ssa#EG819142	Glutaminyl-peptide cyclotransferase-like	- 1.11	- 1.49	0.0455

Results were obtained by two-way ANOVA analysis (p < 0.05; fold change cut-off of 1.2) with Benjamini-Hochberg multiple testing correction. Transcripts (109 probes; 18% unknowns) are arranged by categories of biological function and, within these, by p-value. Indicated are also the probe names and the expression ratio determined separately between families with high vs low total lipid level, for each n-3 LC-PUFA grouping. Percentages of distribution of genes within each category do not include non-annotated probes, those representing the same gene or with a miscellaneous function.

Therefore, the results did not identify lipid metabolism pathways that might underlie differences in flesh n-3 LC-PUFA composition between families. However, previous studies demonstrated that hepatic metabolism genes typically show only low fold changes, even when comparing highly contrasting nutritional compositions (e.g., inclusion of 100% FO *versus* 100% VO in diets [8,9]), compared to immune response genes that tend to be regulated with higher magnitudes of change [10]. Hence, nutritional data such as the present data have been analysed previously without multiple testing correction and this was found to result in relevant biological interpretations, when validated by reverse transcription real time quantitative PCR (RT-qPCR) [9,11]. For this reason, we examined the significant effects of 'n-3 LC-PUFA' without the correction, and from within the list containing 1951 features (significance level, 0.05; fold change cut-off, 1.2), we identified and categorized all 48 lipid metabolism transcripts present (Table 4). An effect on cholesterol metabolism was apparent for the factor 'n-3 LC-PUFA', with several genes of the biosynthesis pathway and its regulation being down-regulated in fish with a high n-3 LC-PUFA phenotype. In addition, glycerophospholipid synthesis, lipid hydrolysis and eicosanoid synthesis and metabolism were also affected, while other genes were associated with lipid and fatty acid transport, fatty acid synthesis and regulation of lipid metabolism.

**Table 4:** Lipid metabolism genes differentially expressed in the liver of fish having high or low flesh n-3 LC-PUFA contents

**Probe name**	**Gene**	**High/Low LC-PUFA**		**p- value**
		**L Lipid (LH/LL)**	**H Lipid (HH/HL)**	
***Cholesterol biosynthesis, metabolism and regulation***				
Omy#S15288895	Sterol-C4-methyl oxidase-like	1.84	1.32	0.0033
Ssa#STIR00031_3	7-dehydrocholesterol reductase (*7dchr*)	- 1.55	- 1.48	0.0036
Ssa#S30286041	7-dehydrocholesterol reductase (*7dchr*)	- 1.48	- 1.42	0.0120
Omy#CF752841	Sterol regulatory element-binding transcription factor 2 (*srebp2*)	- 1.71	- 1.31	0.0170
Ssa#TC102141	Cytochrome P450, family 27, subfamily A, polypeptide 1	- 3.97	- 1.04	0.0192
Ssa#STIR16974	7-dehydrocholesterol reductase (*7dchr*)	- 1.46	- 1.31	0.0195
Ssa#AM402497	Hydroxymethylglutaryl-CoA synthase 1	- 2.39	1.21	0.0199
Ssa#STIR00031_4	7-dehydrocholesterol reductase (*7dchr*)	- 1.41	- 1.46	0.0212
Ssa#STIR00098_4	Isopentenyl-diphosphate delta isomerise (*ipi*)	- 2.12	- 1.15	0.0228
Ssa#S18867829	7-dehydrocholesterol reductase (*7dchr*)	- 1.33	- 1.25	0.0228
Ssa#DY741343	Lanosterol 14-alpha demethylase	- 1.42	- 1.28	0.0346
Omy#S22913656	Acetoacetyl-CoA synthetase	- 1.77	- 2.52	0.0358
Ssa#STIR00033_3	Mevalonate kinase (*mev*)	- 1.54	- 1.08	0.0393
Ssa#CA064135	Vigilin	2.80	- 1.22	0.0423
Ssa#DW582478	Cytochrome P450, family 8, subfamily B, polypeptide 1	- 1.26	- 1.50	0.0449
Ssa#STIR00102_3	Squalene epoxidase	- 2.11	- 1.36	0.0468
***Glycerophospholipid synthesis***				
Ssa#STIR39152_S	Lipid phosphate phosphohydrolase 2 (*lpp2*)	1.20	1.19	0.0177
Ssa#KSS4003	Chka protein	1.18	1.53	0.0264
Ssa#S35538062_S	Monoacylglycerol O-acyltransferase 1 (*mgat*)	1.20	1.51	0.0303
Ssa#S31963704	Diacylglycerol O-acyltransferase homolog 2	1.41	1.02	0.0358
Ssa#S48418830	Phosphatidylglycerophosphate synthase 1	- 1.52	- 1.72	0.0369
***Fatty acid synthesis***				
Ssa#KSS4155	Trans-2-enoyl-CoA reductase, mitochondrial precursor	- 1.26	- 1.25	0.0440
**Intracellular fatty acid transport**				
Ssa#S35585414	Acyl-CoA-binding protein	10.47	1.65	0.0081
Ssa#DY703528	Fatty acid-binding protein, intestinal	- 1.25	- 1.46	0.0331
Ssa#CB509140	Fatty acid-binding protein, intestinal	- 1.20	- 1.35	0.0375
Ssa#STIR04578	Fatty acid-binding protein, heart	1.22	1.47	0.0401
***Lipid transport***				
Ssa#CK898816	Low density lipoprotein receptor-related protein 1 (*lrp1*)	- 1.14	- 1.39	0.0341
Ssa#S32008850	Apolipoprotein A-IV precursor (*apoa4a*)	- 1.43	- 1.01	0.0377
Omy#BX318293	Low density lipoprotein receptor-related protein 1 (*lrp1*)	- 3.61	- 1.67	0.0404
Ssa#S18866963	Apolipoprotein A-IV precursor (*apoa4b*)	- 1.39	- 1.43	0.0428
***Lipid hydrolysis***				
Ssa#S18887340	Acyl-coenzyme A thioesterase 5	1.64	1.18	0.0007
Ssa#STIR02708	Isoamyl acetate-hydrolyzing esterase 1 homolog	1.54	1.12	0.0013
Ssa#DW007099	Similar to patatin-like phospholipase domain containing 7	- 3.75	- 2.37	0.0070
Ssa#S31963297	Acyl-CoA thioesterase 11	1.35	1.38	0.0097
Ssa#STIR07750	N-acylsphingosine amidohydrolase 1	- 1.14	- 1.34	0.0122
Ssa#STIR05034	Isoamyl acetate-hydrolyzing esterase 1 homolog	1.74	1.01	0.0208
Omy#CA366823	Acyl-coenzyme A thioesterase 3	1.33	2.17	0.0248
Ssa#STIR22551	Lipoprotein lipase (*lpl*)	1.31	1.31	0.0346
***Eicosanoid synthesis and metabolism***				
Ssa#TC68569	Thromboxane-A synthase (*thas*)	- 1.33	- 1.67	0.0269
Ssa#TC110080	Phospholipase A2 (*pla2g4*)	1.26	1.08	0.0296
Omy#TC147730	Prostaglandin I2 (prostacyclin) synthase (*ptgis*)	- 2.53	- 4.25	0.0349
Ssa#S35581706	15-hydroxyprostaglandin dehydrogenase	- 1.51	- 1.09	0.0400
Ssa#EG930234	Arachidonate 5-lipoxygenase (*alox5*)	1.91	1.55	0.0449
***Regulation of lipid metabolism***				
Ssa#TC112002	Retinoid X receptor beta	1.00	- 19.09	0.0000
Ssa#KSS2129	Adiponectin receptor protein 1	- 1.30	- 1.31	0.0098
Ssa#CA056493	Angiopoietin-like 6	- 1.63	- 1.07	0.0290
Ssa#S35490606	Adiponectin, C1Q and collagen domain containing, like	1.34	1.66	0.0441
Ssa#S18888608	Adiponectin receptor protein 1	- 1.38	- 1.40	0.0451

Results were obtained by two-way ANOVA analysis (p < 0.05; fold change cut-off of 1.2) without multiple testing correction (n = 1951 total features). Transcripts are arranged by functional categories and, within these, by p-value. Indicated are also the probe names and the expression ratio between families determined separately for each total lipid level.

### Validation of results by RT-qPCR

To validate the microarray analysis results, expression of selected genes was quantified by RT-qPCR. These genes were chosen from lipid metabolism pathways that were more highly affected by the factor 'n-3 LC-PUFA', and also included immune response genes, which was the category most highly affected by both 'n-3 LC-PUFA' and 'total lipid' factors. In addition, the expression of two fatty acyl desaturases (*Δ5fad and Δ6fad*) and one elongase (*elovl2*), which are typically responsive to dietary levels of n-3 LC-PUFA were also determined. The LC-PUFA biosynthesis pathway was not identified by the microarray analysis as being differentially expressed in families with different n-3 LC-PUFA flesh contents but, given the potential importance of this pathway in determining n-3 PUFA phenotypes, we specifically aimed to verify this result. The RT-qPCR results confirmed that genes involved in LC-PUFA biosynthesis were not differentially expressed in families with higher and lower levels of n-3 LC-PUFA (Table 5). Furthermore, the RT-qPCR results confirmed significant down-regulation of genes involved in hepatic cholesterol biosynthesis, such as isopentenyl-diphosphate isomerase (*ipi*), 7-dehydrocholesterol reductase (*7dchr*) and sterol regulatory element-binding protein 2 (*srebp2*) in families containing higher levels of n-3 LC-PUFA in their flesh although this was only observed when this phenotype was also associated with low lipid level, except for *7dchr*, which was significantly down-regulated irrespective of lipid level. With regards to lipoprotein metabolism (lipid transport) genes, general trends such as the magnitude and direction of change were broadly similar between the microarray and the RT-qPCR analysis for the high versus low n-3 LC-PUFA comparison at low lipid contents, although RT-qPCR results were not significant. In the case of high lipid contents, the match between microarray and RT-qPCR data was less consistent, except for lipoprotein lipase (*lpl*), which was similarly up-regulated albeit non-significantly. Up-regulation of the glycerophospholipid biosynthesis pathway in fish with higher n-3 LC-PUFA contents was also indicated when associated with high lipid levels, significant for monoacylglycerol O-acyltransferase 1 (*mgat*). With regards to the eicosanoid biosynthesis pathway, the microarray results could only be confirmed for arachidonic 5-lipoxygenase (*alox5*). Validation of lipid metabolism genes affected by the 'total lipid' factor (Table 6) confirmed the lower expression of *elovl2* in salmon presenting higher lipid levels in their flesh, independent of LC-PUFA content. Finally, good agreement was found between the microarray and RT-qPCR results for immune response genes in response to both 'n-3 LC-PUFA' (Table 5) and 'total lipid' (Table 6) factors.

**Table 5:** Validation of microarray results and expression of genes of interest in relation to the factor 'n-3 LC-PUFA level'

**Gene**	**Low Lipid LH/LL**		**High Lipid HH/HL**	
	**Microarray**	**RT-qPCR**	**Microarray**	**RT-qPCR**
*LC-PUFA biosynthesis*				
*Δ5fad*		−1.19		1.17
*Δ6fad*		1.13		−1.21
*elovl2*		1.14		1.06
*Cholesterol biosynthesis*				
*ipi*	−2.13	**−3.92**	−1.15	1.32
*mev*	−1.54	−1.51	−1.08	1.06
*7dchr*	−1.33 to −1.54	**−1.47**	−1.25 to −1.47	**−1.34**
*srebp2*	−1.72	**−1.68**	−1.30	1.60
*Lipid transport and lipoprotein metabolism*				
*lrp1*	−1.14 to −3.57	−1.36	−1.39 to −1.67	1.24
*apoA4a*	−1.43	−1.09	1.00	1.32
*apoA4b*	−1.39	−1.48	−1.43	−1.10
*lpl*	1.31	1.23	1.31	1.38
*Glycerophospholipid synthesis*				
*lpp2*	1.20	−1.19	1.19	1.30
*mgat*	1.20	1.04	1.51	1.78
*Eicosanoid biosynthesis*				
*alox5*	1.91	**1.48**	1.55	**1.62**
*pla2g4*	1.26	−1.08	1.08	1.06
*thas*	−1.33	−1.08	−1.67	**1.34**
*ptgis*	−2.53	−1.25	−4.25	1.27
*Immune response*				
*mal*	−22.20	**−3.70**	−25.00	**−5.00**
*ccl13*	4.40	**5.98**	3.00	2.14
*trim25*	5.20	**2.80**	6.70	**2.51**
*lect2*	4.60	1.92	7.10	**7.57**

Values represent the expression ratios between high PUFA / low PUFA, for fish containing either low or high total lipid levels in their flesh, obtained by microarray analysis or RT-qPCR. Expression ratios in bold were significant by REST2008 analysis of RT-qPCR results.

Delta5 and 6 fatty acyl desaturases (*Δ5fad and Δ6fad*); fatty acyl elongase (*elovl2*); isopentenyl-diphosphate isomerase (*ipi*); mevalonate kinase (*mev*); 7-dehydrocholesterol reductase (*7dchr*); sterol regulatory element-binding protein 2 (*srebp2*); low density lipoprotein receptor-related protein 1 (*lrp1*); apolipoprotein A-IV (*apoa4a* and *apoa4b*); lipoprotein lipase (*lpl*); lipid phosphate phosphohydrolase 2 (*lpp2*); monoacylglycerol O-acyltransferase 1 (*mgat*); arachidonate 5-lipoxygenase (*alox5*); phospholipase A2 (*pla2g4*); thromboxane-A synthase (*thas*); prostaglandin I2 (prostacyclin) synthase (*ptgis*); myelin and lymphocyte protein (*mal*); c-c motif chemokine 13 precursor (*ccl13*); tripartite motif-containing protein 25 (*trim25*); leukocyte cell-derived chemotaxin 2 precursor (*lect2*).

**Table 6:** Validation of microarray results and expression of genes of interest in relation to the factor 'Lipid level'

**Gene**	**Low n-3 LC-PUFA HL/LL**		**High n-3 LC-PUFA HH/LH**	
	**Microarray**	**RT-qPCR**	**Microarray**	**RT-qPCR**
*LC-PUFA biosynthesis*				
*Δ5fad*		−1.03		1.35
*Δ6fad*		1.04		−1.32
*elovl2*	−1.28 to −1.41	**−1.51**	−1.82 to −1.97	**−1.62**
*Cholesterol biosynthesis*				
*ipi*		**−3.95**		1.31
*mev*		−1.40		1.14
*7dchr*		−1.01		1.09
*srebp2*		**−2.09**		1.29
*Lipid transport and lipoprotein metabolism*				
*lrp1*		**−1.82**		−1.08
*apoA4a*		**−1.37**		−0.95
*apoA4b*		**−2.43**		**−1.80**
*lpl*		−1.18		−1.05
*Immune response*				
*cfh*	−1.33 to −1.56	−1.24	−1.56 to −1.69	**−1.31**
*trim25*	−5.64	**−2.09**	−4.34	−2.33
*lect2*	3.16	1.23	4.86	**4.84**

Values represent the expression ratios between high lipid / low lipid, for fish containing either low or high n-3 LC-PUFA levels in their flesh, obtained by microarray analysis or RT-qPCR. Expression ratios in bold were significant by REST2008 analysis of RT-qPCR results.

Delta5 and 6 fatty acyl desaturases (*Δ5fad and Δ6fad*); fatty acyl elongase (*elovl2*); isopentenyl-diphosphate isomerase (*ipi*); mevalonate kinase (*mev*); 7-dehydrocholesterol reductase (*7dchr*); sterol regulatory element-binding protein 2 (*srebp2*); low density lipoprotein receptor-related protein 1 (*lrp1*); apolipoprotein A-IV (*apoa4a* and *apoa4b*); lipoprotein lipase (*lpl*); complement factor H precursor (*cfh*); tripartite motif-containing protein 25 (*trim25*); leukocyte cell-derived chemotaxin 2 precursor (*lect2*).

### Genetic evaluations

Subsequent to the dietary trial and microarray analyses, genetic evaluations (estimated breeding values, EBVs) became available for a range of traits upon which the families are under active selection in the breeding program. Given the unexpectedly high preponderance of immune response genes identified by transcriptomic analysis, we investigated associations with traits that could potentially explain the gene expression data. In this respect, one of the most relevant traits was ‘survival to infectious pancreatic necrosis (IPN) virus’, known to be almost entirely controlled by a major QTL [12]. Genetic evaluations included data collected from a freshwater experimental IPN challenge on full-sibs from the same families as the trial fish. Examining the families, selected on their lipid phenotypes, used for transcriptomic analysis it was seen that family HH, containing both high total lipid and high n-3 LC-PUFA flesh contents, also showed a high EBV for survival to IPN (selection differential on a standardized normal distribution = 1.86 standard deviations), contrasting with −0.83 (LL) -0.99 (LH) and −1.28 (HL) for the other families, that could introduce a potential for bias in interpretation of the transcriptomic responses. However, no such imbalance was present in the lower lipid grouping, comparing families LL and LH (additional file 3).

## Discussion

The present study which ascertained lipid profiles of 50 Atlantic salmon families confirmed previous results showing important inter-family variation in the ability to retain n-3 LC-PUFA in the flesh when fish are fed diets with low levels of these fatty acids [7]. Furthermore, even though a high correlation was found between flesh lipid levels and n-3 LC-PUFA contents, families with the same total lipid level varied significantly in n-3 LC-PUFA contents. In the present study we did not examine whether these differences have a genetic basis, as this was established previously [7], but instead aimed to identify molecular pathways whose transcriptional regulation might underlie the phenotypic differences, independent of lipid content.

### LC-PUFA biosynthesis

Differences in flesh n-3 LC-PUFA content in individuals fed the same diet is likely to arise from either selective incorporation and retention of fatty acids supplied by the diet or from biosynthesis from precursors in tissues such as the liver. In the present study we performed a transcriptomic study to identify molecular mechanisms potentially underlying flesh n-3 LC-PUFA phenotypes. Expression of candidate genes of the LC-PUFA biosynthesis pathway were also quantified as there was good evidence that these genes are transcriptionally regulated and that mRNA levels correlate with enzymatic activity of this pathway [13,14], and so this appeared a likely mechanism that required specific investigation. Flesh was the target tissue for analysis of the n-3 LC-PUFA retention trait because salmon accumulate lipid reserves in muscle and this is the main product for human consumption, and so its composition will affect the health-promoting properties of salmon. However, hepatic tissue was analyzed for effects on gene expression since the production of both LC-PUFA and the lipoproteins that transport them to the tissues takes place mainly in the liver [15].

The transcriptomic analysis revealed few effects of the n-3 LC-PUFA factor on metabolism in general and, in particular, a lack of effect on lipid metabolism genes, when the statistical analysis employed multiple testing correction. However, this correction is typically not used when examining effects of diet and genetic background on metabolic genes, which tend to show subtle, but physiologically relevant, changes [9,11,16]. Without multiple testing correction we were able to identify pathways of lipid metabolism that might be altered in response to this factor, although a clear mechanism for the observed inter-family differences in n-3 LC-PUFA content was not identified. Potential effects on lipid transport and lipoprotein metabolism were indicated by the presence of two apolipoprotein A4 transcripts (*apoa4a* and *b*), a low density lipoprotein (LDL) receptor-related protein (*lrp1*) and a lipoprotein lipase (*lpl*) transcript in the microarray analysis, albeit these were not validated by RT-qPCR. In contrast, the RT-qPCR results clearly confirmed that the flesh n-3 LC-PUFA phenotype cannot be explained by transcriptional modulation of genes of LC-PUFA biosynthesis and so other mechanisms must be in operation. One hypothesis might be that phenotypic differences between families originates from the presence of different alleles of fatty acyl desaturases and/or elongases encoding proteins with altered biological activity or specificity, as described for the nematode *Caenorhabditis elegans*[17].

### Effects of n-3 LC-PUFA flesh contents on hepatic cholesterol biosynthesis

Within the lipid metabolism genes that were differentially expressed in the liver between fish showing higher or lower n-3 LC-PUFA contents in flesh, the category of cholesterol biosynthesis and its regulation was the most apparent, based on the number of probes for interrelated genes present in this list, all with coordinated regulation indicating reduced cholesterol biosynthesis in salmon having higher flesh n-3 LC-PUFA. In addition, and inferred by the magnitude of change (i.e., fold-changes), effects were more pronounced in fish containing lower flesh lipid levels. These results were confirmed by quantifying the expression of three enzymes catalyzing steps in cholesterol biosynthesis (*mev*, *ipi* and *7dchr*) as well as *srebp2*, a transcription factor that regulates cholesterol synthesis [18]. Furthermore, the RT-qPCR analysis indicated that this regulation was only associated with lower flesh lipid levels given that in the high lipid group only *7dchr* was down-regulated. Therefore, this experiment confirmed previous studies suggesting an association between flesh adiposity and n-3 LC-PUFA in the regulation of cholesterol biosynthesis in Atlantic salmon families, with lean fish showing a higher responsiveness to n-3 LC-PUFA [8]. However, an important novel outcome of the present study was the demonstration that the previous results were not solely a consequence of a higher dietary intake of cholesterol supplied by a FO diet in contrast to a VO diet [11] but also resulted from higher incorporation and increased tissue levels of n-3 LC-PUFA. The likely explanation for these results is the role of n-3 LC-PUFA as regulators of gene transcription, including some implicated in cholesterol biosynthesis, mediated by *srebp2*[18-20]. Nonetheless, the mechanism for why this response was only observed when associated with low flesh lipid levels requires clarification. Recent studies showed that lean humans are also more responsive, in terms of plasma lipid and lipoprotein composition, to cholesterol-reducing diets containing lower levels of saturated fatty acids and cholesterol than obese individuals, and several mechanisms have been proposed to explain this [21]. In the present case, the absolute, rather than the relative, level of n-3 LC-PUFA may be the determinant factor affecting gene transcription and, in the high lipid group, absolute levels of these fatty acids might have been sufficiently high to repress cholesterol biosynthesis genes, even at lower relative n-3 LC-PUFA contents (i.e., group HL). This hypothesis is supported by the RT-qPCR analysis comparing the families with regards to lipid level, HL/LL and HH/LH. In the HL/LL comparison, contrasting absolute n-3 LC-PUFA levels of 427 *versus* 363 mg/100 g flesh, there was down-regulation of both *ipi* and *srebp2* (−3.95 and −2.09, respectively), whereas comparison of the families HH/LH, containing 554 *versus* 468 mg/100 g flesh, showed no difference in the expression of the genes. Similarly, genes involved in lipoprotein metabolism, which are also regulated by LC-PUFA through different mechanisms [20], also showed more significant changes when comparing fatter and leaner salmon with lower LC-PUFA levels, indicating that a similar regulatory mechanism might occur. Therefore, the present study is consistent with previous work identifying cholesterol and lipoprotein metabolism as pathways significantly and differentially affected by n-3 LC-PUFA depending on flesh adiposity [8].

### Effects of total lipid level on lipid metabolism

Lipid level significantly affected expression of lipid metabolism genes, although effects were still relatively small (8% of all genes assigned to a biological function category). A noteworthy result was the down-regulation of *elovl2* (confirmed by RT-qPCR) in salmon presenting higher flesh lipid, independent of LC-PUFA content. Elovl2 has substrate specificity towards LC-PUFA and is highly responsive to dietary n-3 LC-PUFA levels in salmon [22]. However, the expression of this gene is often co-ordinately regulated with other genes of LC-PUFA biosynthesis, such as *Δ5fad* and *Δ6fad*[9], which was not the case here. Hence, the biological significance of this result is not clear and may indicate other roles of *elovl2* in lipid metabolism. For instance, an association between overexpression of *elovl2* and enhanced triacylglycerol synthesis and lipid droplet accumulation, as well as induction of PPARγ target genes, was shown in mouse preadipocyte cell lines [23]. In addition, *elovl2* was up-regulated in the liver transcriptome of rats with nephrotic syndrome, a condition characterized by hyperlipidemia [24]. Elovl2 was only recently characterized in salmon [22], and this is the first indication of an association between its expression and lipid accumulation in a non-mammalian vertebrate, with results suggesting that increased lipid level in salmon flesh repressed *elovl2* expression independent of n-3 LC-PUFA level although this requires further investigation. Another gene down-regulated at higher lipid levels was a mitochondrial acyl carrier protein, involved in acyl transfer steps, including roles in fatty acid synthesis and functioning of the electron transport chain [25], which could conceptually be responding to similar regulatory mechanisms affecting *elovl2*. In contrast, stearoyl-CoA desaturase, responsible for the synthesis of monounsaturated fatty acids from saturated precursors, was up-regulated in salmon with higher flesh lipid levels. This gene was positively correlated with fat accumulation in bovine skeletal muscle [26], consistent with up-regulation in salmon families with increased fat stores.

### Possible association between flesh n-3 LC-PUFA contents and immune response

The predominance of immune response genes responding to total lipid level and, particularly, n-3 LC-PUFA contents in salmon flesh was unexpected. This was a true over-representation as GO enrichment analysis enabled identification of several GO terms related to regulation of immune and inflammatory responses in relation to the total lipid factor. However, as mentioned above, the transcriptomic comparison, although balanced for total lipid, was not balanced for viral disease resistance (specifically IPN in this case) and, as a consequence, higher contrast between families was imposed on the high lipid group (families HL and HH) due to the fortuitous selection of family HH presenting a much higher viral resistance EBV. Nonetheless, if family HH biased the results of the two-way ANOVA we would expect a preponderance of immune-related genes to occur only when comparing these two families, presenting higher and lower flesh n-3 LC-PUFA contents at the higher lipid level. In order to assess this, t-tests were performed comparing separately the higher versus lower n-3 LC-PUFA families at each total lipid level, i.e., LH/LL and HH/HL. A Venn diagram contrasting the two t-test significant lists was then performed and when analyzing the genes that were similarly affected by n-3 LC-PUFA contents at both higher and lower total lipid level, a similar preponderance (33%) of immune response genes was observed (Additional file 4). Finally, examination of the fold changes of immune-related genes, indicating magnitude of effects, between families with higher and lower contents of n-3 LC-PUFA at either higher or lower total lipid levels (Tables 2 and 5), showed no clear evidence of the effect being more marked for the high lipid comparison, which is what would be expected if results were caused simply by inclusion of family HH in the transcriptomic analysis.

Hence, there is evidence to suggest that there may be some correlation between flesh n-3 LC-PUFA contents and immune response in the families analysed. An anti-inflammatory role of n-3 LC-PUFA is well established in mammals and fish [27-29]. Immune cells are typically rich in arachidonic acid (ARA), the precursor for eicosanoids with a pro-inflammatory action, whereas EPA and DHA give rise to eicosanoids that are less biologically active, as well as to resolvins and protectins presenting anti-inflammatory properties [30]. Higher incorporation of n-3 LC-PUFA in biological membranes of immune cells can modulate immune responses in several ways [reviewed in [15,30-33]. They alter the production of inflammatory eicosanoid mediators of which they are precursors, directly affect the organization and properties of the immune cell membranes with effects on signalling pathways, phagocytic capacity and antigen presenting capability, and activate transcription of various genes involved in inflammatory responses. Therefore, families with higher tissue levels of n-3 LC-PUFA may show differential expression of immune response and inflammation-related genes, as well as of genes involved in signalling and regulation of transcription (as observed in the present study). Furthermore, although liver is chiefly a metabolic organ, it has other physiological functions including removal of pathogens and antigens from the blood and modulation of immune responses, as well as the production of inflammatory mediators [34,35].

Related to the above, microarray analysis revealed the presence of several genes that intervene in eicosanoid synthesis and metabolism including phospholipase A_2_ (*pla2*), arachidonate 5-lipoxygenase (*alox5*), thromboxane-A synthase (*thas*), prostaglandin I_2_ synthase (*ptgis*) and 15-hydroxyprostaglandin dehydrogenase [36]. However, RT-qPCR only confirmed up-regulation of hepatic *alox5* in families presenting higher flesh n-3 LC-PUFA and, given that *alox5* acts on LC-PUFA of both n-3 and n-6 series and that ARA levels generally accompanied the n-3 LC-PUFA phenotype (Table 1), it cannot be ascertained whether this transcript was responding to higher levels of membrane ARA or EPA and hence if it would result in increased pro-inflammatory 4-series, or less potent 5-series, leukotrienes [37].

The immune response genes whose expression was correlated with 'n-3 LC-PUFA' are mainly involved in the modulation of inflammatory processes and innate immune response to pathogens, which are particularly important in fish species and that can be easily compromised in aquaculture conditions [38]. We could speculate that the changes in expression may give enhanced protection from inflammation or pathological conditions in fish with higher n-3 LC-PUFA in their tissues. Up-regulation associated with high flesh n-3 LC-PUFA was noted in expression of NACHT domain containing protein, tripartite motif-containing protein 25 (*trim25*), c-c motif chemokine 13 precursor (*ccl13*), leukocyte cell-derived chemotaxin 2 precursor (*lect2*), tissue factor pathway inhibitor a, pentraxin and cathepsin K. In contrast, down-regulation in the high n-3 LC-PUFA families was observed for MHC class I (mostly in the high total lipid group), and for myelin and lymphocyte protein (*mal*). NACHT domain containing proteins are pathogen-sensing molecules (recognizing intracellular pathogen-associated molecular patterns – PAMPs) implicated in early host defence, inflammation and innate immune signalling pathways in mammals [39], by activating transcription of MHC class II and the apoptotic pathway. The *trim25* protein is involved in antiviral innate immune responses through activation of signalling pathways leading to production of interferons and in teleost cells TRIM genes are induced in response to viral infections [40,41]. The *ccl13* (also known as monocyte chemotactic protein 4) and *lect2* proteins are both involved in inflammation, having roles in attracting monocytes and T lymphocytes in tissues exposed to exogenous pathogens, and have neutrophil chemotactic function [42,43]. Expression of *lect2* was increased in fish liver and spleen after bacterial infections [43]. Tissue factor pathway inhibitor inhibits the initial reactions of the blood coagulation cascade and modulates cell proliferation, and may protect vascular tissue in inflammatory conditions in mammals [44]. Cathepsin K mediates immune responses in cells, having a critical role in signalling events proximal to the Toll-like receptor 9 (TLR9) that has a fundamental role in pathogen recognition (recognizing PAMPs) and activation of mammalian innate immunity [45]. Finally, pentraxins are pattern recognition proteins of the innate immune system that play a role in the acute phase response, activating complement pathways to clear pathogens in both mammals and fish [46,47]. In this case, up-regulation of pentraxin in salmon with higher n-3 LC-PUFA in their flesh was only observed with high lipid levels. Similarly, down-regulation of the MHC class I transcript was observed only in the high lipid group. In mammalian studies, high LC-PUFA contents (EPA, DHA and ARA) reduced cell surface expression of MHC I, decreasing antigen presentation and altering T-cell signalling [34,35]. Therefore, the high IPN resistance genotype observed in family HH in later genetic evaluations of the families could potentially involve effects on both the complement pathway and T-cell mediated immunity, and involve co- or post-translational modification of proteins by N-linked glycosylation through up-regulation of dolichyl-diphosphooligosaccharide-protein glycosyltransferase subunit 4 (Table 2; [48]). Given the high economic impact of IPN in salmonid culture, identification of genes potentially involved in the progression of the disease using transcriptomic approaches is already in progress [49]. Finally, down-regulation of *mal*, associated with T-cell differentiation and signal transduction [50], was observed at higher n-3 LC-PUFA levels.

As mentioned above, several immune response-related genes were also affected by the total lipid factor with results validated by RT-qPCR. However, we cannot exclude the possibility that this results from the strong correlation between total lipid levels and absolute LC-PUFA contents, which makes it difficult to dissociate both factors.

## Conclusions

It has been demonstrated earlier that LC-PUFA flesh content is a highly heritable trait [7], but the present study has shown that the underlying mechanisms do not appear to involve changes in the expression of lipid metabolism genes, including the LC-PUFA biosynthesis pathway. Other possible mechanisms, such as alleles with different biological activity, require investigation. The present study revealed an association between flesh adiposity and n-3 LC-PUFA in the regulation of cholesterol biosynthesis, which was down-regulated by higher n-3 LC-PUFA levels but only in the lean families. This response was not caused by dietary factors, given that the fish were all fed the same VO-based diet, and is most likely explained by variation in tissue n-3 LC-PUFA levels, regulating transcription of cholesterol metabolism genes through *srebp2*. Furthermore, the transcriptional repression of these genes may be sensitive to the absolute levels of these fatty acids in the tissues, which could explain the lack of regulation when comparing the families containing higher flesh lipid levels. It is likely that n-3 LC-PUFA exert similar roles in regulation of gene expression in fish as in mammals and, furthermore, fish might be a useful model to study important relationships between genetics, diet, adiposity/obesity and lipoprotein/cholesterol metabolism. However, unexpected differences were found in the expression of genes implicated in the modulation of inflammatory processes and innate immune response between families differing in lipid composition, both in terms of total lipid level and, particularly, n-3 LC-PUFA contents. Although the evidence is generally circumstantial it is important to clarify this association if flesh n-3 LC-PUFA level is included as a trait for genetic selection in Atlantic salmon breeding programmes. If such a relationship is confirmed, the data suggest that the underlying mechanism might involve anti-inflammatory actions of tissue n-3 LC-PUFA on the eicosanoid biosynthesis pathway (particularly affecting the lipoxygenase pathway), although direct effects through regulation of transcription of immune genes or more indirectly through changes in architecture and properties of immune cell membranes are also possible.

## Methods

### Feeding trial and sampling

Fifty full-sib families selected from the 200 broodstock families of the Landcatch Natural Selection (LNS) Atlantic salmon breeding program (2005-strip year-group) were specifically selected for the feeding trial. On the basis of parental genetic evaluations, 25 high flesh lipid contrasting with 25 low flesh lipid families were identified, and 35 fish (initial weight, ~100 g) from each family were transferred and grown in communal seawater pens (Marine Harvest, Ardnish, Scotland). All fish were tagged with electronic transponders (PIT tags) to allow family identification while rearing in a common environment. After acclimation, the fish were grown for 12 weeks on the same low FM/high VO diet (Nutreco ARC, Stavanger, Norway) containing 25% FM and 44% plant meals and a VO blend including rapeseed oil/palm oil/camelina oil (2.5:1.5:1). At the end of the trial (378 g average weight), flesh samples (Norwegian Quality Cut) were collected, frozen on dry ice and stored at −20°C until lipid analysis. Liver samples were also taken and stored at −70°C for subsequent molecular analyses.

### Lipid analysis and choice of families for transcriptomic comparisons

The 50 selected families were screened for their ability to retain and/or synthesize n-3 LC-PUFA when fed a low FM/high VO diet. De-boned and skinned flesh samples were combined into 3 pools per family for lipid analysis. Total lipids were extracted and determined gravimetrically from 1–2 g of pooled flesh [51]. Fatty acid methyl esters (FAME) were prepared by acid-catalyzed transesterification of total lipids [52]. Following purification, FAME were separated and quantified by gas–liquid chromatography as described in [9]. These data were used to select four families for transcriptomic analysis: two with equivalent high levels of lipid ‘H’, and two with equivalent low levels of lipid ‘L’. Within each level of total lipid, two families with significantly contrasting (p < 0.05 on Student’s t-test; Graphpad Prism™, version 4.0, Graphpad Software, San Diego, CA) relative n-3 LC-PUFA levels (similarly termed H and L) were identified (Table 1).

### RNA extraction and purification

Hepatic tissue (200 mg) from ten individuals per family was rapidly homogenized in 2 ml TRI Reagent (Ambion, Applied Biosystems, Warrington, U.K.). Total RNA was isolated, following manufacturer’s instructions, and RNA quality and quantity was assessed by gel electrophoresis and spectrophotometry (NanoDrop ND-1000, Thermo Scientific, Wilmington, U.S.A.), respectively. Equal amounts (50 μg) of total RNA were pooled from two individuals to produce five biological replicates per family, which were further purified by mini spin-column purification (RNeasy Mini Kit, Qiagen, Crawly, U.K.).

### Microarray hybridization and analysis

A custom-made Atlantic salmon oligoarray with 44 K features per array on a four-array-per-slide format (Agilent Technologies, Cheshire, U.K.), with experimental features printed singly was used [described more fully in [53]. The probes were co-designed at the Institute of Aquaculture, University of Stirling, U.K. and Nofima, Norway, with array design available in the EBI ArrayExpress database (http://www.ebi.ac.uk/arrayexpress/arrays/browse.html) under accession number A-MEXP-2065. The features were mainly derived from a core set of Atlantic salmon Unigenes (NCBI) supplemented with other unique cDNAs derived from Genbank and the Atlantic Salmon Gene Index (http://compbio.dfci.harvard.edu/tgi/tgipage.html). Probe annotations were derived from Blastx comparisons across four protein databases, as detailed elsewhere [54]. The entire experiment comprised 20 hybridizations (5 slides): 4 groups (families) × 5 biological replicates (pools of 2 individuals each).

Indirect labelling was employed in preparing the microarray targets, as described in detail previously [8]. Antisense amplified RNA (aRNA) was produced from 500 ng of each total RNA purification reaction using the Amino Allyl MessageAmpTM II aRNA Amplification Kit (Ambion, Applied Biosystems), following the manufacturer’s methodology followed by Cy3 or Cy5 fluor (PA23001 or PA25001, GE HealthCare) incorporation through a dye-coupling reaction.

The hybridizations were performed using SureHyb hybridisation chambers (Agilent) in a DNA Microarray Hybridisation Oven (Agilent). Sample order was semi-randomized, with one replicate per experimental group being loaded into each slide. Each biological replicate pool was co-hybridized in a two-dye experiment with a single pooled reference sample. This pooled reference comprised equal quantitites of aRNA from all 20 biological replicate pools. Microarry manufacturer’s instructions were followed. Briefly, for each hybridization, 825 ng of Cy3-labelled experimental biological replicate and Cy5-labelled reference pool were combined. A fragmentation master mix containing 10× blocking agent (Agilent), 25× fragmentation buffer (Agilent) and nuclease-free water, was dispensed into the Cy-dyes mix. After incubating in the dark at 60°C for 30 mins, 2× GE Hybridization buffer (pre-heated to 37°C; Agilent) was added, contents gently mixed, spun at 16 K *g* for 1 min and finally kept on ice until loaded onto the microarray slides. Hybridization was carried out in the oven rotator (Agilent) at 65°C and 10 rpm for 17 h. Post-hybridization washes were carried out in EasyDipTM Slide staining containers (Canemco Inc., Quebec, Canada). After disassembling the array-gasket sandwiches submersed in wash buffer 1 (Agilent) at room temperature, the microarray slides were incubated in wash buffer 1 for 1 min at 31°C in a Stuart Orbital Incubator S150 rotating at 150 rpm, and then a further 1 min at 31°C at 150 rpm in wash buffer 2 (Agilent). A final dip in wash buffer 2 at room temperature was performed, after which the slides were dried by centrifugation (500 xg for 6 mins) and kept in a desiccator and in the dark until scanned, the same day.

Scanning was performed at 5 μm resolution using an Axon GenePix 4200AL Scanner (MDS Analytical Technologies, Wokingham, Berkshire, U.K.). Laser power was kept constant (50%) and the “auto PMT” function within the acquisition software (v.4) was enabled to adjust PMT for each channel such that less than 0.1% of features were saturated and that the mean intensity ratio of the Cy3 and Cy5 signals was close to one. Agilent Feature Extraction Software (v 9.5) was used to identify features and extract fluorescence intensity values from the resultant TIF images. Analysis of the intensity values was performed in the GeneSpring GX version 11 analysis platform (Agilent Technologies, Wokingham, Berkshire, U.K.). All intensity values <0.1 were set to equal 0.1 followed by a Lowess normalization. After removing control features, four quality filtering steps were carried out sequentially using a range of quality control metrics produced by the Agilent Feature Extraction software to remove features that were saturated, non uniform, population outliers and spots non-significantly different from background. This gave a final list of 32,566 probes that were eligible for statistical analysis. Experimental annotation complied fully with minimum information about a microarray experiment (MIAME) guidelines [55]. The experimental hybridizations and further methodological details are archived on the EBI ArrayExpress database under accession number E-TABM-1204.

Normalized and quality-filtered fluorescence intensity data was analysed in GeneSpring GX v11 by two-way ANOVA, which examined the explanatory power of the variables ‘total lipid’ and ‘n-3 LC-PUFA’ and the interaction between the two, at a significance level of 0.05 and expression ratio (i.e., fold change) cut-off of 1.2. Two sets of analysis were performed, with or without Benjamini-Hochberg multiple testing correction. In the set with multiple testing correction, GO enrichment analysis was performed at a significance level of 0.05.

### RT-qPCR

Expression of selected genes found by microarray analysis to be significantly affected by either ‘total lipid’ or ‘n-3 LC-PUFA’ content was quantified by RT-qPCR. In addition, the expression of two fatty acyl desaturases (*Δ5fad* and *Δ6fad*) and one elongase (*elovl2*) that are typically responsive to dietary n-3 LC-PUFA was determined. Primers were designed using Primer3 software (http://biotools.umassmed.edu/bioapps/primer3_www.cgi) (Table 7). Two reference genes, elongation factor-1α (*elf-1α*) and *β-actin*, were also quantified.

**Table 7:** Primers used for RT-qPCR analyses

**Transcript**	**Primer sequence (5’-3’)**	**Fragment**	**Ta**	**Efficiency**	**Accession No.**	**Source**
*Δ5fad*	GTGAATGGGGATCCATAGCA	192 bp	56°C	0,945	AF478472 ^1^	[9]
	AAACGAACGGACAACCAGA					
*Δ6fad_a*	CCCCAGACGTTTGTGTCAG	181 bp	56°C	0,928	AY458652 ^1^	[9]
	CCTGGATTGTTGCTTTGGAT					
*elovl2*	CGGGTACAAAATGTGCTGGT	145 bp	60°C	0,926	TC91192 ^2^	[24]
	TCTGTTTGCCGATAGCCATT					
*ipi*	ACAGCCCTATGGTTATGTGTCATCTC	230 bp	60°C	0,985	CK875291 ^1^	[11]
	CAAGGTGAGGCGAATGTTTGAAC					
*mev*	CCCTTAATCAGGGTCCCAAT	247 bp	60°C	0,910	DW005667 ^1^	[11]
	GGTGCTGGTTGATGTCAATG					
*7dchr*	CTTCTGGAATGAGGCATGGT	230 bp	60°C	0,977	TC99602 ^2^	[11]
	ACAGGTCCTTCTGGTGGTTG					
*srebp2*	GACAGGCACAACACAAGGTG	215 bp	60°C	0,887	DY733476 ^1^	[11]
	CAGCAGGGGTAAGGGTAGGT					
*lrp1*	ACCAACCGCATCTACTGGAC	204 bp	60°C	0,996	CK898816 ^1^	New design
	CAGATTACCAGCCACCCAGT					
*apoA4a*	CCCAAACCAACACCACTCCT	150 bp	60°C	0,997	BT047465 ^1^	New design
	GGTTTATATTTCTCACCCTGCAC					
*apoA4b*	CTCTTGCCCTCTTGATGACTG	154 bp	60°C	0,918	BT047267 ^1^	New design
	TGACTCATCAGAGCCAATTCA					
*lpl*	AGGGCGTTAATCCATGTCAG	223 bp	60°C	0,917	TC84899 ^2^	[8]
	GACCTTTCAAAAGGGCATGA					
*lpp2*	TCCGGAAGAACTCGCAATAC	174 bp	60°C	0,926	NM_001140716 ^1^	[9]
	ACATCACGTCCACCAAGACA					
*mgat*	TTAACCCAAAGATGCTGCAA	157 bp	60°C	0,977	EG824440 ^1^	New design
	CACGCAGTTGTCAGTGGTTT					
*alox5*	TATCTCCCTCTCCCTCAGTCC	155 bp	56°C	0,987	CX727592 ^1^	[57]
	GGTCAGCAGTGCCATCA					
*pla2g4*	GTCGCTGGCTGGAGCTGTGG	138 bp	60°C	0,998	NM_001141333 ^1^	New design
	AGCCCTATGGGCCCTGGTCA					
*thas*	TGTTCACACGGACCTGATTC	150 bp	60°C	0,986	NM_001165312 ^1^	New design
	GACCGGATCGTCATTCTGTT					
*ptgis*	GCGTGTTTGTGGTCATTACG	247 bp	60°C	0,836	GE778709 ^1^	New design
	TTCCCTTAGCAAGGTCTGGA					
*mal*	GGCCTCAGTCAAAGAGGAGA	156 bp	60°C	0,946	NM_001141320 ^1^	New design
	GGGGAGTGCACACTTTAGGA					
*ccl13*	CGAGGATCCCTCTTCAACAA	178 bp	60°C	0,996	EG831431 ^1^	New design
	ATCGTCGACTAGGCAGCAGT					
*trim25*	GCAGGGTCCTATCTCATCCA	215 bp	60°C	0,951	BT048046 ^1^	New design
	GGACTGGACCTTTTTATTCTCTCA					
*lect2*	CTGTGTTGTCAGAGTGCGAGATGGT	150 bp	60°C	0,996	BT050009 ^1^	[58]
	TACACACAATGTCCAGGCCCTGA					
*cfh*	TGTGATGATGGAGAGATGCAG	193 bp	60°C	0,966	TC141997 ^2^	New design
	CAAGCGACAAAGAAACCACA					
Reference genes*						
*elf-1α*	CTGCCCCTCCAGGACGTTTACAA	175 bp	60°C	1.000	AF321836 ^1^	[11]
	CACCGGGCATAGCCGATTCC					
*β-actin*	ACATCAAGGAGAAGCTGTGC	141 bp	56°C	0.939	AF012125 ^1^	[11]
	GACAACGGAACCTCTCGTTA					

^1^ GenBank (http://www.ncbi.nlm.nih.gov/).

^2^ Atlantic salmon Gene Index (http://compbio.dfci.harvard.edu/tgi/).

* geNorm average stability (M value) of reference genes = 0.514 [59].

For RT-qPCR, 2 μg of column-purified total RNA per sample was reverse transcribed into cDNA using the High-Capacity cDNA RT kit (Applied Biosystems, Paisley, U.K.), following manufacturer’s instructions, but using a mixture of the random primers (1.5 μl as supplied) and anchored oligo-dT (0.5 μl at 400 ng/μl, Eurofins MWG Operon, Ebersberg, Germany). Negative controls (containing no enzyme) were performed to check for genomic DNA contamination. A similar amount of cDNA was pooled from all samples and the remaining cDNA was then diluted 20-fold with water. RT-qPCR analysis used relative quantification with the amplification efficiency of the primer pairs being assessed by serial dilutions of the cDNA pool. Amplifications were carried out in duplicate (Quantica, Techne, Cambridge, U.K.) in a final volume of 20 μl containing 5 μl or 2 μl (for more highly expressed genes) diluted (1/20) cDNA, 0.5 μM of each primer (0.4 μM for *lect2*) and 10 μl AbsoluteTM QPCR SYBR® Green mix (ABgene). Amplifications were carried out with a systematic negative control (NTC-non template control, containing no cDNA). The RT-qPCR profiles contained an initial activation step at 95°C for 15 min, followed by 30 to 40 cycles: 15 s at 95°C, 15 s at the specific primer pair annealing temperature (Ta; Table 7) and 15 s at 72°C. After the amplification phase, a melt curve of 0.5°C increments from 75°C to 90°C was performed, enabling confirmation of the amplification of a single product in each reaction. Non-occurrence of primer-dimer formation in the NTC was verified. RT-qPCR product sizes and presence of single bands were checked by agarose gel electrophoresis. Additionally, sequencing of amplicons corresponding to new primer designs enabled the confirmation of identities and presence of single sequences for all genes except for *trim25*, as the sequencing result was of insufficient quality to conclude on the presence of a single gene product, and *lrp1*, for which results were indicative of quantification of a highly similar, recently duplicated, gene.

Results were analyzed by the ΔΔCt method using the relative expression software tool (REST 2009, http://www.gene-quantification.info/), which employs a pair wise fixed reallocation randomization test (10,000 randomizations) with efficiency correction [56], to determine the statistical significance of expression ratios (or gene expression fold-changes) between two treatments.

### Genetic evaluations of traits used in the salmon breeding program

Parental evaluations were confirmed by subsequent analysis of family sibs (at harvest weight, some 1 year after the present study) for a range of traits upon which the breeding program families are under active selection including flesh lipid composition parameters (total lipid) as well as EBVs for weight at harvest, precocious maturation, flesh colour, sealice resistance and resistance to a viral infection (IPN).

## Competing interests

The authors declare that they have no competing interests.

## Author’s contributions

SM performed laboratory analyses and data analysis. DRG was responsible for family selection. JBT supported the microarray analysis. SM wrote the first draft of the manuscript, followed by contributions from remaining authors. SM, JBT and DRT planned and coordinated the research. DRG, JGB and DRT were project leaders. All authors read and approved the final manuscript.

## Supplementary Material

Additional file 1 **Figure S1. **Relationship between total lipid level and n-3 LC-PUFA content. Click here for file

Additional file 2 **Table S1.** Gene Ontology terms showing significant enrichment in the list of features affected by the 'total lipid' factor. Click here for file

Additional file 3 **Figure S2.** Distribution of IPN resistance scores in relation to flesh lipid phenotypes. Click here for file

Additional file 4 **Table S2. **Genes similarly regulated in pair-wise comparisons of families containing H and L n-3 LC-PUFA flesh contents, at each one of the total lipid levels. Click here for file
