# Supplementary material for: Hepatic transcriptome analysis of inter-family variability in flesh n-3 long-chain polyunsaturated fatty acid content in Atlantic salmon
Source: BMC Genomics. 2012 Aug 20;13:410. doi: 10.1186/1471-2164-13-410 (PMC3463449; doi:10.1186/1471-2164-13-410)
Supplement: Additional file 1 — Figure S1. Relationship between total lipid level and n-3 LC-PUFA content. [file 1471-2164-13-410-S1.doc]

**Additional file 1:** **Relationship between total lipid level and n-3 LC-PUFA content.** The graphs show the relationship between total lipid level (g/100g flesh) and n-3 LC-PUFA content as either a relative value (µg/mg lipid) – top graph – or an absolute amount (mg/100g flesh) – bottom graph. Indicated are the correlation coefficients (r) and associated p-values between both lipid composition parameters. Squares around the data points indicate the four families which were used for molecular analysis.
