# Supplementary material for: Hepatic transcriptome analysis of inter-family variability in flesh n-3 long-chain polyunsaturated fatty acid content in Atlantic salmon
Source: BMC Genomics. 2012 Aug 20;13:410. doi: 10.1186/1471-2164-13-410 (PMC3463449; doi:10.1186/1471-2164-13-410)
Supplement: Additional file 2 — Table S1. Gene Ontology terms showing significant enrichment in the list of features affected by the 'total lipid' factor. [file 1471-2164-13-410-S2.doc]

**Additional file 2: Gene Ontology terms showing significant enrichment in the list of features affected by the 'total lipid' factor.** GO analysis was conducted at p<0.05 after two-way ANOVA analysis of the microarray results (p<0.05; fold change cut-off of 1.2; with Benjamini-Hochberg multiple testing correction).

| GO ACCESSION | GO Term | Corrected p-value | % Count in Selection | % Count in Total |
| --- | --- | --- | --- | --- |
| GO:0050778 | positive regulation of immune response | 2,9E-07 | 20 | 0,25 |
| GO:0050776 | regulation of immune response | 2,9E-07 | 20 | 0,28 |
| GO:0002684 | positive regulation of immune system process | 6,2E-07 | 20 | 0,32 |
| GO:0048584 | positive regulation of response to stimulus | 8,7E-07 | 20 | 0,28 |
| GO:0002682 | regulation of immune system process | 2,6E-06 | 20 | 0,38 |
| GO:0048583 | regulation of response to stimulus | 4,9E-05 | 20 | 0,37 |
| GO:0002699 | positive regulation of immune effector process | 1,9E-04 | 16 | 0,08 |
| GO:0002697 | regulation of immune effector process | 4,9E-04 | 16 | 0,10 |
| GO:0006954 | inflammatory response | 9,7E-04 | 24 | 0,40 |
| GO:0001819 | positive regulation of cytokine production | 1,1E-02 | 16 | 0,07 |
| GO:0016614 | oxidoreductase activity, acting on CH-OH group of donors | 1,4E-02 | 30 | 0,63 |
| GO:0006952|GO:0002217|GO:0042829 | defense response | 1,4E-02 | 28 | 0,76 |
| GO:0004616 | phosphogluconate dehydrogenase (decarboxylating) activity | 1,9E-02 | 16 | 0,07 |
| GO:0006739 | NADP metabolic process | 2,0E-02 | 16 | 0,20 |
| GO:0002708 | positive regulation of lymphocyte mediated immunity | 2,6E-02 | 12 | 0,06 |
| GO:0002706 | regulation of lymphocyte mediated immunity | 2,6E-02 | 12 | 0,06 |
| GO:0002705 | positive regulation of leukocyte mediated immunity | 2,6E-02 | 12 | 0,06 |
| GO:0002703 | regulation of leukocyte mediated immunity | 2,6E-02 | 12 | 0,06 |
| GO:0001817 | regulation of cytokine production | 2,7E-02 | 16 | 0,08 |
| GO:0019863 | IgE binding | 3,3E-02 | 12 | 0,04 |
| GO:0006766 | vitamin metabolic process | 3,6E-02 | 18 | 0,48 |
| GO:0002673 | regulation of acute inflammatory response | 4,4E-02 | 12 | 0,05 |
| GO:0002675 | positive regulation of acute inflammatory response | 4,4E-02 | 12 | 0,05 |
| GO:0002712 | regulation of B cell mediated immunity | 4,4E-02 | 12 | 0,05 |
| GO:0002714 | positive regulation of B cell mediated immunity | 4,4E-02 | 12 | 0,05 |
| GO:0002819 | regulation of adaptive immune response | 4,4E-02 | 12 | 0,05 |
| GO:0002821 | positive regulation of adaptive immune response | 4,4E-02 | 12 | 0,05 |
| GO:0002822 | regulation of adaptive immune response based on somatic recombination of immune receptors built from immunoglobulin superfamily domains | 4,4E-02 | 12 | 0,05 |
| GO:0002824 | positive regulation of adaptive immune response based on somatic recombination of immune receptors built from immunoglobulin superfamily domains | 4,4E-02 | 12 | 0,05 |
| GO:0002861 | regulation of inflammatory response to antigenic stimulus | 4,4E-02 | 12 | 0,05 |
| GO:0002863 | positive regulation of inflammatory response to antigenic stimulus | 4,4E-02 | 12 | 0,05 |
| GO:0002864 | regulation of acute inflammatory response to antigenic stimulus | 4,4E-02 | 12 | 0,05 |
| GO:0002866 | positive regulation of acute inflammatory response to antigenic stimulus | 4,4E-02 | 12 | 0,05 |
| GO:0002883 | regulation of hypersensitivity | 4,4E-02 | 12 | 0,05 |
| GO:0002885 | positive regulation of hypersensitivity | 4,4E-02 | 12 | 0,05 |
| GO:0002889 | regulation of immunoglobulin mediated immune response | 4,4E-02 | 12 | 0,05 |
| GO:0002891 | positive regulation of immunoglobulin mediated immune response | 4,4E-02 | 12 | 0,05 |
| GO:0006098 | pentose-phosphate shunt | 4,4E-02 | 16 | 0,20 |
| GO:0006740 | NADPH regeneration | 4,4E-02 | 16 | 0,20 |
| GO:0006769 | nicotinamide metabolic process | 4,4E-02 | 16 | 0,23 |
| GO:0016616 | oxidoreductase activity, acting on the CH-OH group of donors, NAD or NADP as acceptor | 4,4E-02 | 30 | 0,61 |
| GO:0019814 | immunoglobulin complex | 4,4E-02 | 14 | 0,08 |
| GO:0019882|GO:0030333 | antigen processing and presentation | 4,4E-02 | 14 | 0,24 |
