# Supplementary material for: Hepatic transcriptome analysis of inter-family variability in flesh n-3 long-chain polyunsaturated fatty acid content in Atlantic salmon
Source: BMC Genomics. 2012 Aug 20;13:410. doi: 10.1186/1471-2164-13-410 (PMC3463449; doi:10.1186/1471-2164-13-410)
Supplement: Additional file 3 — Figure S2. Distribution of IPN resistance scores in relation to flesh lipid phenotypes. [file 1471-2164-13-410-S3.doc]

**Additional file 3: Distribution of IPN resistance scores in relation to flesh lipid phenotypes.** The plots show estimated breeding values (EBV) for IPN survival (logit transformation of data where 0-dead and 1-alive), assessed by a freshwater experimental challenge, in relation to either total lipid level (g/100g flesh) (upper panel) or absolute n-3 LC-PUFA content (mg/100g flesh) (lower panel) of all families in the selection program. Indicated (squares around the data points) are the four families used for molecular analysis.
