# Supplementary material for: Hepatic transcriptome analysis of inter-family variability in flesh n-3 long-chain polyunsaturated fatty acid content in Atlantic salmon
Source: BMC Genomics. 2012 Aug 20;13:410. doi: 10.1186/1471-2164-13-410 (PMC3463449; doi:10.1186/1471-2164-13-410)
Supplement: Additional file 4 — Table S2. Genes similarly regulated in pair-wise comparisons of families containing H and L n-3 LC-PUFA flesh contents, at each one of the total lipid levels. [file 1471-2164-13-410-S4.doc]

**Additional file 4: Genes similarly regulated in pair-wise comparisons of families containing H and L n-3 LC-PUFA flesh contents, at each one of the total lipid levels.** Two t-tests (unpaired unequal variance, Welch, asymptotic) with no multiple testing correction, at p < 0.05 and fold change >1.2, were performed, each comparing the two families presenting higher and lower n-3 LC-PUFA contents at either one of the total lipid levels: HH/HL and LH/LL. This returned a list of 1604 or 1305 entities for the comparison in the low or high total lipid grouping, respectively. A Venn diagram was subsequently performed with these two lists and a Venn list containing only the genes that were present in both t-test lists was retrieved. The Venn list contained 149 entities but, of these, only 64 entities presented a similar regulation (up- or down-regulated in both lists). These were then categorized according to their biological function (9% unknowns) and are presented in the table. Indicated are also the probe names, the expression ratio for HH/HL and LH/LL comparisons, and their respective p-values. Percent representation of genes by category of biological function (after removing unknown and genes with miscellaneous function, as well as repeated probes for the same gene) is also shown.

| **Probe name** | **Gene** | **Hi/Lo LC-PUFA** | | **p- value** | |
| --- | --- | --- | --- | --- | --- |
|  |  | **H Lipid**  **HH/HL** | **L Lipid**  **LH/LL** | **H Lipid**  **HH/HL** | **L Lipid**  **LH/LL** |
|  |  |  |  |  |  |
| ***Metabolism (9%)*** | | | | |  |
|  | | | | |  |
| *Energy metabolism (3%)* | | | | |  |
| Ssa#S31991256 | Isocitrate dehydrogenase 3 (nad+) | - 1.7 | - 1.9 | 0.0233 | 0.0046 |
|  |  |  |  |  |  |
| *Xenobiotic and oxidant metabolism (6%)* | | | | |  |
| Ssa#STIR00161_3 | Cytochrome P450 1A | 1.8 | 2.5 | 0.0045 | 0.0047 |
| Con_CANDS_13 | Cytochrome P450 1A | 1.4 | 2.0 | 0.0071 | 0.0101 |
| Ssa#S18892279 | Cytochrome P450 1A | 1.4 | 1.8 | 0.0112 | 0.0041 |
| Ssa#STIR05682 | Peroxiredoxin 5 | 1.3 | 1.4 | 0.0122 | 0.0207 |
| Ssa#STIR00161_4 | Cytochrome P450 1A | 1.5 | 2.1 | 0.0210 | 0.0145 |
| Ssa#STIR00161_2 | Cytochrome P450 1A | 1.4 | 1.9 | 0.0286 | 0.0019 |
|  |  |  |  |  |  |
| ***Translation (6%)*** | | | | |  |
| Ssa#KSS2048 | Mitochondrial ribosomal protein S33 | - 1.8 | - 1.9 | 0.0028 | 0.0171 |
| Ssa#S30281303 | Mitochondrial 28S ribosomal protein S33 | - 1.7 | - 1.7 | 0.0105 | 0.0395 |
| Ssa#S18867312 | Ribonuclease UK114 | 1.4 | 1.4 | 0.0132 | 0.0030 |
|  |  |  |  |  |  |
| ***Regulation of transcription (28%)*** | | | | |  |
| Ssa#S35481301 | TIP120 protein | 3.4 | 3.1 | 0.0019 | 0.0471 |
| Ssa#S35664778 | Zinc finger protein 41 | 1.5 | 1.5 | 0.0037 | 0.0245 |
| Omy#S18104058 | Zinc finger protein 235 | 2.3 | 4.1 | 0.0208 | 0.0006 |
| Ssa#S31964227 | Similar to GTF2I repeat domain containing 2 | 2.5 | 3.3 | 0.0224 | 0.0161 |
| Ssa#CN181117 | U2-associated protein SR140 (140 kDa Ser/Arg-rich domain protein) | 3.1 | 4.4 | 0.0242 | 0.0068 |
| Ssa#S18880068 | SET and MYND domain-containing protein 3 | 1.8 | 2.1 | 0.0244 | 0.0313 |
| Ssa#S35500885 | Transcription factor 15 | 3.7 | 3.5 | 0.0262 | 0.0355 |
| Omy#TC171818 | Zinc finger CCCH domain-containing protein 14 | 1.3 | 1.5 | 0.0279 | 0.0287 |
| Ssa#S31985656 | Prolactin regulatory element-binding protein | 2.4 | 4.4 | 0.0329 | 0.0116 |
| Ssa#TC97482 | CREB binding protein transcript variant | 2.9 | 3.6 | 0.0355 | 0.0342 |
|  | | | | |  |
| *Signalling and protein modification (22%)* | | | | |  |
| Ssa#S35552908 | Tyrosine 3-monooxygenase/tryptophan 5-monooxygenase activation protein, epsilon polypeptide | - 2.1 | - 1.6 | 0.0037 | 0.0395 |
| Ssa#CA055651 | Serine/threonine-protein phosphatase 6 | 13.0 | - 6.5 | 0.0091 | 0.0193 |
| Ssa#STIR07369 | Serine threonine protein kinase raf1 | 1.3 | 1.8 | 0.0118 | 0.0033 |
| Omy#TC167859 | Regulator of calcineurin family member | - 1.4 | - 1.4 | 0.0139 | 0.0418 |
| Ssa#S30241573 | Tyrosine 3-monooxygenase/tryptophan 5-monooxygenase activation protein, epsilon polypeptide | - 1.5 | 1.3 | 0.0187 | 0.0499 |
| Ssa#STIR31840 | Sphingomyelin phosphodiesterase acid transcript variant 1 | 2.3 | 2.1 | 0.0238 | 0.0003 |
| Ssa#S23659857 | Sphingomyelin phosphodiesterase acid transcript variant 1 | 2.0 | 1.6 | 0.0278 | 0.0100 |
| Ssa#STIR23254 | Serine/threonine-protein phosphatase 2A regulatory subunit B subunit gamma | 1.2 | 1.7 | 0.0286 | 0.0053 |
| Ssa#S35559485 | P2Y purinoceptor 1 | 1.4 | 1.4 | 0.0414 | 0.0046 |
| Ssa#STIR11129 | Ras-related protein Rap-2b precursor putative | - 4.5 | - 5.8 | 0.0460 | 0.0071 |
|  |  |  |  |  |  |
| ***Immune response (36%)*** | | | | |  |
| Ssa#S35581943 | Myelin and lymphocyte protein | - 25.0 | - 22.2 | 0.0004 | 0.0059 |
| Ssa#S35558945 | Tripartite motif-containing protein 25 | 6.5 | 4.8 | 0.0011 | 0.0240 |
| Ssa#S35536179 | novel NACHT domain containing protein | 1.8 | 2.4 | 0.0016 | 0.0018 |
| Ssa#S35516341 | Tripartite motif-containing protein 25 | 6.7 | 5.2 | 0.0023 | 0.0044 |
| Ssa#STIR02298 | C-C motif chemokine 13 precursor putative | 3.0 | 4.4 | 0.0029 | 0.0015 |
| Ssa#TC70262 | Cathepsin K | 2.3 | 1.4 | 0.0050 | 0.0153 |
| Ssa#KSS3969 | Leukocyte cell-derived chemotaxin 2 precursor | 7.1 | 4.6 | 0.0071 | 0.0008 |
| Ssa#S30265745 | Bleomycin hydrolase | - 1.6 | - 1.6 | 0.0127 | 0.0056 |
| Ssa#TC93758 | BOLA class I histocompatibility antigen, alpha chain BL3-7 precursor | - 9.0 | - 18.6 | 0.0133 | 0.0473 |
| Ssa#STIR21287 | Solute carrier family 30 (zinc transporter)member 7 | 1.5 | 2.1 | 0.0134 | 0.0006 |
| Ssa#STIR29382 | Rhamnose-binding lectin WCL1 | 1.4 | 1.5 | 0.0165 | 0.0375 |
| Ssa#S29966058 | MHC class I antigen | 8.4 | 1.9 | 0.0171 | 0.0457 |
| Ssa#S35566535 | CD4 | 1.6 | 1.6 | 0.0266 | 0.0040 |
| Ssa#S19108411 | Rhamnose-binding lectin WCL1 | 1.3 | 1.5 | 0.0343 | 0.0111 |
| Ssa#S35509469 | GTPase IMAP family member 7 | 1.7 | 4.0 | 0.0417 | 0.0043 |
|  | | | | |  |
| Miscellaneous/unknown function | | | | |  |
| Ssa#STIR09736 | Transmembrane protein 42 | 1.9 | 2.3 | 0.0004 | 0.0005 |
| Ssa#STIR02934 | Ripply2 protein | - 2.0 | - 2.0 | 0.0009 | 0.0242 |
| Ssa#DW552272 | Usher syndrome 1C (autosomal recessive, severe) | - 16.2 | - 8.1 | 0.0037 | 0.0308 |
| Ssa#S18842295 | Alveolin | 4.6 | 1.7 | 0.0081 | 0.0001 |
| Ssa#TC110572 | Usher syndrome 1C (autosomal recessive, severe) | - 9.1 | - 3.6 | 0.0093 | 0.0415 |
| Omy#CX031967 | X-ray repair complementing defective repair in Chinese hamster cells 3 | - 2.1 | - 3.1 | 0.0174 | 0.0280 |
| Ssa#S30242447 | Serine protease HTRA1 | - 2.3 | - 3.8 | 0.0281 | 0.0361 |
| Omy#S15322430 | Oxidative stress induced growth inhibitor 1 | - 1.4 | - 1.3 | 0.0312 | 0.0489 |
| Ssa#CB517995 | Neuroligin 3 | 3.8 | 4.3 | 0.0341 | 0.0344 |
| Ssa#DY726487 | WD repeat domain 44 | - 3.8 | - 3.5 | 0.0428 | 0.0098 |
| Ssa#S35564744 | Claudin 10 | 2.1 | 4.6 | 0.0473 | 0.0191 |
| Omy#S19712209 | N-acylglucosamine 2-epimerase | - 2.7 | - 6.0 | 0.0482 | 0.0382 |
| Ssa#STIR11043 | Golgi autoantigen, golgin subfamily b, macrogolgin (with transmembrane signal), 1 (GOLGB1) | - 1.4 | - 1.8 | 0.0500 | 0.0118 |
